# Spatial distribution and risk factors of *Schistosoma haematobium* and hookworm infections among schoolchildren in Kwale, Kenya

**DOI:** 10.1371/journal.pntd.0005872

**Published:** 2017-09-01

**Authors:** Evans Asena Chadeka, Sachiyo Nagi, Toshihiko Sunahara, Ngetich Benard Cheruiyot, Felix Bahati, Yuriko Ozeki, Manabu Inoue, Mayuko Osada-Oka, Mayuko Okabe, Yukio Hirayama, Mwatasa Changoma, Keishi Adachi, Faith Mwende, Mihoko Kikuchi, Risa Nakamura, Yombo Dan Justin Kalenda, Satoshi Kaneko, Kenji Hirayama, Masaaki Shimada, Yoshio Ichinose, Sammy M. Njenga, Sohkichi Matsumoto, Shinjiro Hamano

**Affiliations:** 1 Leading Program, Graduate School of Biomedical Sciences, Nagasaki University, Nagasaki, Japan; 2 Department of Parasitology, Institute of Tropical Medicine (NEKKEN), Nagasaki University, Nagasaki, Japan; 3 The Joint Usage/Research Center on Tropical Disease, Institute of Tropical Medicine (NEKKEN), Nagasaki University, Nagasaki, Japan; 4 Department of Vector Ecology and Environment, Institute of Tropical Medicine (NEKKEN), Nagasaki University, Nagasaki, Japan; 5 Nagasaki University, Kenya Research Station, NUITM-KEMRI Project, Nairobi, Kenya; 6 Department of Bacteriology, Niigata University School of Medicine, Niigata, Japan; 7 Department of Bacteriology and Virology, Osaka-City University Graduate School of Medicine, Osaka, Japan; 8 Food Hygiene and Environmental Health Division of Applied Life Science, Graduate School of Life and Environmental Sciences, Kyoto Prefectural University, Kyoto, Japan; 9 Department of Immunology, National Institute of Infectious Diseases, Tokyo, Japan; 10 Department of Immunology, Yamaguchi University Graduate School of Medicine, Ube, Japan; 11 Eastern and Southern Africa Centre of International Parasite Control (ESACIPAC), Kenya Medical Research Institute (KEMRI), Nairobi, Kenya; 12 Department of Immunogenetics, Institute of Tropical Medicine (NEKKEN), Nagasaki University, Nagasaki, Japan; 13 Department of Eco-Epidemiology, Institute of Tropical Medicine (NEKKEN), Nagasaki University, Nagasaki, Japan; University of Florida, UNITED STATES

## Abstract

**Background:**

Large-scale schistosomiasis control programs are implemented in regions with diverse social and economic environments. A key epidemiological feature of schistosomiasis is its small-scale heterogeneity. Locally profiling disease dynamics including risk factors associated with its transmission is essential for designing appropriate control programs. To determine spatial distribution of schistosomiasis and its drivers, we examined schoolchildren in Kwale, Kenya.

**Methodology/Principal findings:**

We conducted a cross-sectional study of 368 schoolchildren from six primary schools. Soil-transmitted helminths and *Schistosoma mansoni* eggs in stool were evaluated by the Kato-Katz method. We measured the intensity of *Schistosoma haematobium* infection by urine filtration. The geometrical mean intensity of *S*. *haematobium* was 3.1 eggs/10 ml urine (school range, 1.4–9.2). The hookworm geometric mean intensity was 3.2 eggs/g feces (school range, 0–17.4). Heterogeneity in the intensity of *S*. *haematobium* and hookworm infections was evident in the study area. To identify factors associated with the intensity of helminth infections, we utilized negative binomial generalized linear mixed models. The intensity of *S*. *haematobium* infection was associated with religion and socioeconomic status (SES), while that of hookworm infection was related to SES, sex, distance to river and history of anthelmintic treatment.

**Conclusions/Significance:**

Both *S*. *haematobium* and hookworm infections showed micro-geographical heterogeneities in this Kwale community. To confirm and explain our observation of high *S*. *haematobium* risk among Muslims, further extensive investigations are necessary. The observed small scale clustering of the *S*. *haematobium* and hookworm infections might imply less uniform strategies even at finer scale for efficient utilization of limited resources.

## Author summary

The World Health Organization is spearheading the war on neglected tropical diseases, including helminth infections, by encouraging its member states to intensify control efforts. This call has recently been answered in most endemic regions of helminthiasis and governments are scaling up chemotherapy-based control programs in collaboration with private and public partners. However, it is necessary to clearly understand factors driving local transmission dynamics of helminth infections to design effective control programs. Here, we conducted a cross-sectional survey of 368 primary schoolchildren in Kwale, Kenya, and identified factors associated with the intensity of *Schistosoma haematobium* and hookworm infections. The negative binomial generalized linear mixed model showed the intensity of *S*. *haematobium* infection was much higher among Muslims and schoolchildren from low socioeconomic status households. High intensity of hookworm infection was associated with sex, SES, distance to river and history of anthelmintic treatment. Our findings demonstrate considering social and cultural drivers of NTDs could be beneficial in designing of efficient control programs and expediting NTDs control.

## Introduction

Schistosomiasis and soil-transmitted helminthiases are among neglected tropical diseases targeted for control by the World Health Organization (WHO) [1]. Globally, soil-transmitted helminths (STHs), such as hookworms (*Ancylostoma duodenale* and *Necator americanus*), *Ascaris lumbricoides* and *Trichuris trichiura*, infect 1.5 billion people [2]. By 2014, 258 million individuals were estimated to be suffering from schistosomiasis, which is endemic in 78 countries worldwide. In Kenya, approximately 17.4 million people are at risk of schistosomiasis [3] and approximately 9.1 million Kenyans are in danger of soil-transmitted helminthiases [4].

Two schistosome pathogens dominant in Kenya are *Schistosoma haematobium*, which causes urogenital schistosomiasis, and S*chistosoma mansoni*, which is responsible for intestinal schistosomiasis [4]. Disease distribution chiefly depends on the presence of *Bulinus* spp. and *Biomphalaria* spp. as intermediate host snails for *S*. *haematobium* and *S*. *mansoni*, respectively [5]. Along the Kenyan coast, schistosomiasis is almost entirely caused by *S*. *haematobium*. The constant high temperature along the coast restricts the proliferation of *Biomphalaria* spp. host snails in the area [6]. Streams, seasonal pools, quarry pits and drainage canals are primary habitats for *Bulinus* spp. along the Kenyan coast [7].

Apart from distribution of intermediate host snails, sanitation and human contact with infested water play a significant role in schistosomiasis transmission [8,9]. The intensity of infection is influenced by water contact frequency and duration in infested water [8]. Factors such as age, gender, occupation, female household head’s education level, religion, SES and house location can influence a person’s contact with infested water [10–13]. Therefore, dynamics of helminth infection can, to some extent, be viewed as a result of the behavior and livelihoods of individuals in the context of their physical, economic, social and cultural environments.

Small-scale spatial heterogeneity is one of the most striking features of schistosomiasis from an epidemiological point of view and is chiefly due to locally determined factors [13–17]. Past studies have also linked persistence of schistosomiasis and soil-transmitted helminthiasis in endemic regions to low socioeconomic status (SES). Low SES can result in lack of access to safe water and improved sanitation in addition to poor hygiene practices [18,19].

Identifying local epidemiological drivers of helminthiasis in endemic areas is necessary to generate vital data for improving current control programs toward achieving maximum benefits. This study, therefore, determined factors associated with the intensity of *S*. *haematobium* and hookworm infections among schoolchildren in Kwale, Kenya.

## Methods

### Study area

**Fig 1:**
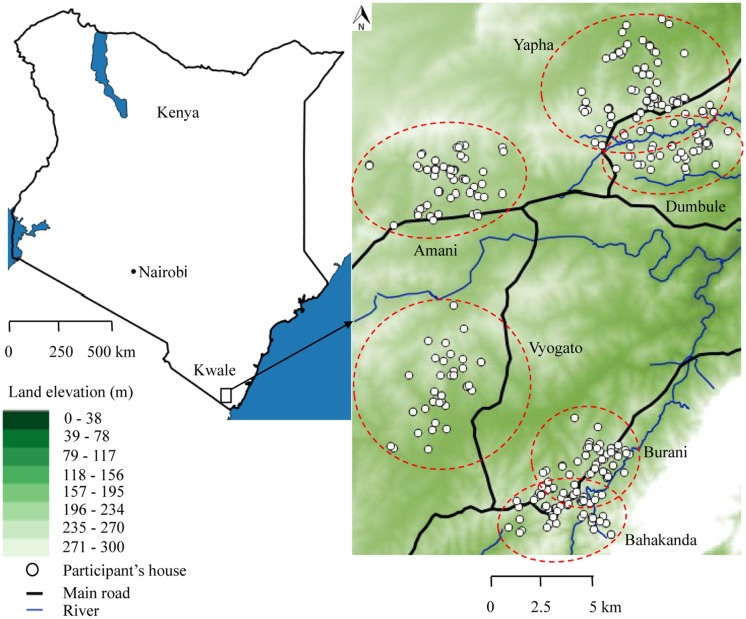
Map of the study area, Kwale, Kenya. Dotted red circles indicate the catchment area from which children attend each school. The position of the participants’ houses is indicated by white circles. The river network is shown by blue lines while the main road is represented by black lines. Altitude (meters): highest, white background; lowest, dark green background.

The study was carried out in Kwale, a rural setting located on the south coast of Kenya (Fig 1). There is an established Health and Demographic Surveillance System (HDSS) in Kwale which covers an area of 384.9 km^2^ with 7,617 households and 42,585 inhabitants. HDSS Kwale lies between latitudes 4°17′S and 4°5′S and longitudes 39°15′E and 39°29′E [20]. Compared to other counties in Kenya, Kwale is among the poorest. More than half of the population does not have access to improved sanitation [21]. Residents of Kwale engage in farming as their primary economic activity for subsistence. The two largest religions are Islam and Christianity.

The net primary school enrolment rate is about 80% which is lower than the national average [22]. Kwale benefited from deworming exercise of the Kenya National Program for Elimination of Lymphatic Filariasis. Individuals aged 2 years and over received a single-dose of albendazole and diethylcarbamazine citrate in 2003, 2005 and 2008. The respective treatment coverage was 77%, 76% and 62.8% [23,24]. The Kenya National School-Based Deworming Programme (NSBDP) to control schistosome and STH infections was launched in 2009 where 3.6 million school aged children were dewormed with albendazole in endemic regions. In 2012, the NSBDP was scaled up and albendazole and praziquantel were co-administered to school children in Kwale County in 2013 and 2014. During the year 2015 only albendazole was administered in Kwale County due to logistical challenges experienced by NSBDP in the country [25–27].

### Study design

A cross-sectional study was conducted from January to March 2012. Our study targeted schoolchildren in class 4. Only full grade primary schools were included in this study. There were 40 primary schools in HDSS Kwale, of which 10 were private schools as of January 2012 (Data manager HDSS Kwale, self-report). Twenty-three schools with 1,502 children in class 4 met the inclusion criteria. Since the prevalence of helminthiasis in the study area was unknown, we set it at 50%. Precision and design effect (α = 0.1) were set at 5% and 3%, respectively. Based on these parameters, a sample of 270 children was deemed sufficient for this study. The average size of class 4 in the eligible schools was 47 pupils; with the assumption of a 95% response rate, six schools were adequate for this study. Random cluster sampling of six schools (Fig 1) was performed using R statistical software version 2.13.1 [28]. Ninety-two percent of parents/guardians consented and consequently, 427 children were recruited in the study.

### Questionnaire

Trained interviewers gathered demographic and socioeconomic data from parents/guardians in home settings using a pretested questionnaire. A SES index was constructed based on main floor, wall and roof material of the house. Additionally, we included number of household members sharing a sleeping room and land size. Other components considered for SES were possession of: solar panel, bicycle, radio and mobile phone. Principal component analysis of wealth related variables was conducted in SPSS version 17 [29]. We created a wealth quintile and categorized participants into “Most poor” “very poor,” “poor,” “less poor” and “least poor” groups. Generation of wealth index by PCA is detailed in S1 File. We also gathered data on female household head’s education level. The question on education level had five categories: “none,” “incomplete primary,” “complete primary,” “secondary level” and “at least college level.” This was later categorized as “none/incomplete primary” and “above primary” since majority of participants did not complete primary school. Household religious affiliation was categorized as “Christian,” “Muslim” or “Atheist”. The interviewers verified the age of the children during home visits by cross-checking official birth certificates or baptism cards. Household geo-coordinates were recorded using a handheld global positioning system unit (Garmin eTrex H, Deutschland, Garmin International, Germany). The water contact behavior and shoe wearing practices of the children were assessed through a questionnaire administered to the children at school. Shoe wearing habit was recorded as “always” or “never.” For water contact frequency, the children were asked how often they bathed or washed in the river: “daily”, “3–6 times per week”, “1–2 times per week” or “never”.

### Stool and urine examination for helminth infections

A day before parasitological screening commenced, participating children were issued stool containers. The research team instructed them on how to collect a portion of their stool in the morning on the next day. The study group distributed urine sample containers to participants on the actual screening day between 09:00 and 13:00 hours. We collected both fecal and urine samples for 3 consecutive days. The laboratory staff labeled specimen containers with a unique code assigned to each child. *S*. *mansoni* and STH infections were examined by the Kato-Katz fecal thick smear technique for stool [30]. Briefly, thick fecal smears prepared using 41.7 mg plastic templates were observed within 1 hour using a light microscope to detect and quantify hookworm eggs. The slides were left to clear within 24 hours for identification of *S*. *mansoni*, *T*. *trichiura* and *A*. *lumbricoides* eggs. We multiplied the number of eggs observed by 24 to express infection intensity as the number of eggs per gram of feces (EPG).

For *S*. *haematobium* assessment, 10 ml of urine was aliquoted using disposable syringes and filtered through a polycarbonate filter membrane. The filters measured 25 mm in diameter with a pore size of 12 μm (Whatman, Kent, UK). The urine was filtered at the sample collection sites. The filtrates were placed on labeled microscope glass slides and stored in slide boxes for subsequent analysis under a light microscope. We expressed the intensity of *S*. *haematobium* infection as the number of eggs detected per 10 ml of urine. Our team examined urine on 3 succeeding days to prevent misdiagnosis due to day-to-day variation in egg excretion. Arithmetic mean for the three slides, both for stool and urine, was used to express infection status of each child. An individual was deemed to be *S*. *haematobium* positive if at least one egg was observed on microscopic examination of urine on either day. For school infection intensity, geometric mean was obtained using the n+1 transformation for a series of egg outputs including zero. We categorized the extent of *S*. *haematobium* infection intensity as light (1–49 eggs/10 ml urine) or heavy (≥50 eggs/10 ml urine). Hookworm infection was categorized as light (1–1,999 EPG), moderate (2,000–3,999 EPG) and heavy (≥4,000 EPG). *T*. *trichiura* infection was categorized as light (1–999 EPG), moderate (1,000–9,999 EPG) and heavy (≥10,000 EPG) based on WHO guidelines [31].

### Data analysis

Data were entered into Microsoft Excel 2007 spreadsheets (Microsoft Corp., Redmond, WA, USA) and exported to the statistical package R version 3.2.4 where all statistical analyses were performed [28]. For the final analyses, we included 368 children with complete parasitological and questionnaire data. Since intensity yields morbidity details, unlike prevalence, we tested the association between the intensity of helminth infections and fixed factors. The intensity of helminth infections was expressed as the arithmetic mean of number of slides examined per child. For school mean intensity, we showed the intensity of helminth infections as the geometric mean. Infection intensity is a statistic that measures the estimation of worm numbers per person. Due to the over dispersion of egg counts, such data are well described using the negative binomial probability model [32].

To identify factors associated with the intensity of *S*. *haematobium* and hookworm infections, we fitted negative binomial generalized linear models (NB-GLM) using MASS library for bivariate analysis. For multivariate analysis, we employed the glmmADMB library of the R statistical package [28] to fit negative binomial generalized linear mixed models (NB-GLMM). School variable was included in the NB-GLMM as a random factor to control for the influence of living conditions. For factors associated with the intensity of *S*. *haematobium* infection, the response (i.e., dependent variable) was the average count of *S*. *haematobium* eggs/10 ml urine for each child. Fixed factors (covariates) included age, sex, SES, female household head’s education level, last deworming, bathing/washing in river, house distance from the river, religion and school as a random factor (to control for variability in living conditions). To identify factors associated with the intensity of hookworm infection, the mean hookworm EPG for each child was the response variable. Covariates included age, sex, SES, female household head’s education level, last deworming, religion, shoe wearing habit and school as a random factor. A map of the spatial distribution of the intensity of *S*. *haematobium* infection was developed using QGIS version 2.12.3 [33]. The shortest distance from a participant’s house to the river was measured using the QGIS software. Spatial clustering of the intensity of *S*. *haematobium* and hookworm infections was determined by SaTScan software version 9.4 [34]. To detect high and low clusters we applied normal model to the log (N+1) transformed egg count.

### Ethical consideration

The scientific steering committee and the ethical review board of Kenya Medical Research Institute (SSC No. 2084) authorized this study. The ethical review board of Nagasaki University, Institute of Tropical Medicine, Japan (No. 140829127) also approved this study. Before the commencement of field activities, meetings were held with parents/guardians, school administrators and teachers to discuss the purpose and procedures of the study. We also informed relevant district education and health officers of the research. Parents/guardians consented in writing while children assented to the study before enrollment. On completion of sample analysis, a clinician treated all children infected with schistosomes with 40 mg/kg of praziquantel and those infected with STHs with 400 mg of albendazole in the six schools according to WHO guidelines [31].

## Results

### Sociodemographic characteristics of study subjects

The age range of the children was 8–18 years with a median of 12 years. There were 186 girls (50.5%) and 182 boys (49.5%). Over half (59.8%) of the children were from poor or worse off SES households. A majority (86.1%) of female household heads had not completed primary school education. Islam was the most frequently reported religion (78.5%) in the study area. Table 1 shows characteristics of the study participants. The median shortest distance from participants’ houses to the river was 1,295 meters (range, 33–6,249 meters). Approximately one-third (32.1%) of the children had a daily river water contact. Over half (52.2%) of the children did not wear shoes while outdoors.

**Table 1:** Potential risk factors for *Schistosoma haematobium* and hookworm infections.

Variable		Number (%)
**Age in years**	Median (Range)	12 (8–18)
**Sex**	Girls	186 (50.5)
	Boys	182 (49.5)
**Socio-economic status**	Least poor	72 (19.6)
	Less poor	76 (20.7)
	Poor	77 (20.9)
	Very poor	69 (18.8)
	Most poor	74 (20.1)
**School**	Burani	60 (16.3)
	Vyogato	39 (10.6)
	Bahakanda	71 (19.3)
	Dumbule	45 (12.2)
	Amani	58 (15.8)
	Yapha	95 (25.8)
**Female household heads education level**	Above primary school	51 (13.9)
	None or incomplete primary	317 (86.1)
**Last deworming**	Within a year	115 (42.9)
	Over a year	253 (57.1)
**Bathing/washing in river**	Never	104 (28.4)
	1–2 times a week	69 (18.6)
	3–6 times a week	77 (20.9)
	Daily	118 (32.1)
**Distance to the river**	Range	33–6249 meters
	Median	1295 meters
**Religion**	Christian	79 (21.5)
	Islam	289 (78.5)
**Shoes wearing habit**	Regularly	176 (47.8)
	Never	192 (52.2)

### Prevalence and intensity of *S*. *haematobium* and STH infections

The overall prevalence of at least one helminth infection was 46.2% (95% CI: 41.1–51.3), ranging from 14.7% to 80.0% in the six schools. As indicated in Table 2, the prevalence of *S*. *haematobium*, hookworm and *T*. *trichiura* infection was 33.2% (95% CI: 28.3–38.0), 26.1% (95% CI: 21.6–30.6) and 1.6% (95% CI: 0.3–2.9), respectively. We did not observe any cases of *S*. *mansoni* and *A*. *lumbricoides* infections.

**Table 2:** Number (%) of schoolchildren infected with three parasite species in Kwale, Kenya.

Parasite	Overall n = 368 Prevalence (95% CI)	School						P
		Burani	Vyogato	Bahakanda	Dumbule	Amani	Yapha	
		(n = 60)	(n = 39)	(n = 71)	(n = 45)	(n = 58)	(n = 95)	
*Schistosomes*								
*S*. *haematobium*^1^	33.2 (28.3–38.0)	37 (61.7%)	13 (33.3%)	24 (33.8%)	16 (35.6%)	21 (36.2%)	11 (11.6%)	<0.001
*Light*	19.8 (15.8–23.9)	19 (31.7%)	5 (12.8%)	14 (19.7%)	14 (31.1%)	13 (22.4%)	8 (8.4%)	
*Heavy*	13.3 (9.8–16.8)	18 (30.0%)	8 (20.5%)	10 (14.1%)	2 (4.4%)	8 (13.8%)	3 (3.2%)	
Mean intensity (Eggs/10 ml) ^2^	3.1	9.2	3.4	3.0	2.3	3.7	1.4	
*S*. *mansoni*	0							
STHs								
Hookworm^3^	26.1 (21.6–30.6)	34 (56.7%)	19 (48.7%)	37 (52.1%)	3 (6.7%)	0 (0%)	3 (3.2%)	<0.001
*Light*	24.7 (20.3–29.1)	31 (51.7%)	19 (48.7%)	35 (49.3%)	3 (6.7%)	—	3 (3.2%)	
*Moderate*	1.1 (0.02–2.1)	2 (3.3%)	0 (0%)	2 (2.8%)	0 (0%)	—	0 (0%)	
*Heavy*	0.3 (-0.2–0.8)	1 (1.7%)	0 (0%)	0 (0%)	0 (0%)	—	0 (0%)	
Mean intensity (EPG) ^2^	3.2	17.4	6.2	10.5	1.2	0	1.1	
*T*. *trichiura*^4^	1.6 (0.3–2.9)	3 (5.0%)	0 (0%)	3 (4.2%)	0 (0%)	0 (0%)	0 (0%)	0.0355**
*A*. *lumbricoides*	0							
At least one helminth	46.2 (41.1–51.3)	48 (80.0%)	25 (64.1%)	45 (63.4%)	17 (37.8%)	21 (36.2%)	14 (14.7%)	<0.001

^1^ Light: 1–49 eggs/10 ml urine; heavy: ≥50 eggs/10 ml urine.

^2^ Mean intensity obtained by geometric mean.

^3^ Light: 1–999 EPG; moderate: 1,000–3,999 EPG; heavy: ≥4,000 EPG.

^4^ All cases had light infection (1–999 EPG).

* Prevalence comparison among schools by Fisher’s test.

The geometric mean egg count for *S*. *haematobium* was 2.0 eggs/10 ml urine (range, 0.4–8.0 eggs/10 ml urine) in the six schools. Among children infected with *S*. *haematobium*, 13.3% (95% CI: 9.8–16.8) showed heavy egg numbers in their urine. The geometric mean of hookworm eggs was 2.2 EPG (range, 0–16.5 EPG). The majority of hookworm cases were light infections. All five positive cases for *T*. *trichiura* were light infections.

### Spatial distribution and factors associated with the intensity of *S*. *haematobium* infection

In bivariate analysis without controlling for school effects, the NB-GLM revealed the intensity of *S*. *haematobium* infection to be associated with SES, religion and last deworming. In reference to the least poor, the infection intensity was high in the very poor and less poor categories. Participants affiliated to Islam were more intensely infected than Christians. At school level, Islam was associated with high infection risk except in Vyogato. The children who were dewormed more than one year prior to the study had lower infection intensity compared to those dewormed within a year’s time. Details of bivariate analysis results are shown in Table 3.

**Table 3:** Bivariate negative binomial generalized linear model (NB-GLM) for intensity of *S*. *haematobium* infection among schoolchildren in Kwale, Kenya.

Parameter		Estimate	Std. Error	Z value	Pr(> | Z |)
Age	(8–18 years)	-0.03279	0.13398	-0.245	0.8066
Sex	Boys	0.724	0.453	1.6	0.11
Hookworm infection	Positive	0.4864	0.5192	0.937	0.349
SES* (*Reference = Least poor)	Less poor	1.1182	0.694	1.611	0.1071
	Poor	-0.0117	0.6919	-0.017	0.9865
	Very poor	1.6744	0.7109	2.355	**0.0185**
	Most poor	-0.7034	0.6988	-1.007	0.3142
Female head education	None or incomplete primary	0.1816	0.659	0.276	0.783
Last deworming	Over a year ago	-1.4142	0.4802	-2.945	**0.003**
Bathing in river/dam** (** Reference = Never)	1–2 times a week	0.19004	1.04227	0.182	0.855
	3–6 times a week	0.02145	1.28162	0.017	0.987
	Daily	-0.44261	0.51483	-0.86	0.39
Distance to the river	Far	-1.76E-02	4.55E-01	-0.039	0.969
Religion	Muslim	1.8128	0.545	3.326	**0.0008**
Latrine	Present	0.05178	0.47899	0.108	0.914
Drinking water source*** (***Reference = River)	Well	0.8545	0.6929	1.1233	0.217
	Spring	-0.8306	2.5248	-0.329	0.742
	Tap	0.1658	0.693	0.239	0.811
Shoes wearing habit	Never	0.7003	0.4533	1.545	0.122

On inclusion of school in the NB-GLMM as a random factor, the effects of last deworming became nonsignificant. High intensity of *S*. *haematobium* infection was associated with very poor *P* = 0.0208 and Muslim *P* = 0.0011 (Table 4).

**Table 4:** Negative binomial generalized linear mixed model (NB-GLMM) for intensity of *S*. *haematobium* infection among schoolchildren in Kwale, Kenya.

Parameter		Estimate	Std. Error	Z value	Pr(> | Z |)
Age	8–18 years	0.0989	0.136	0.73	0.4675
Sex	Boys	0.61	0.464	1.31	0.1885
SES* (*Reference = Least poor)	Less poor	0.395	0.759	0.52	0.6023
	Poor	-0.247	0.673	-0.37	0.7142
	Very poor	1.77	0.766	2.31	**0.0208**
	Most poor	-0.135	0.887	-0.15	0.8792
Last deworming	Over a year ago	-0.176	0.536	-0.33	0.7424
Religion	Muslim	2.403	0.738	3.26	**0.0011**

**Fig 2:**
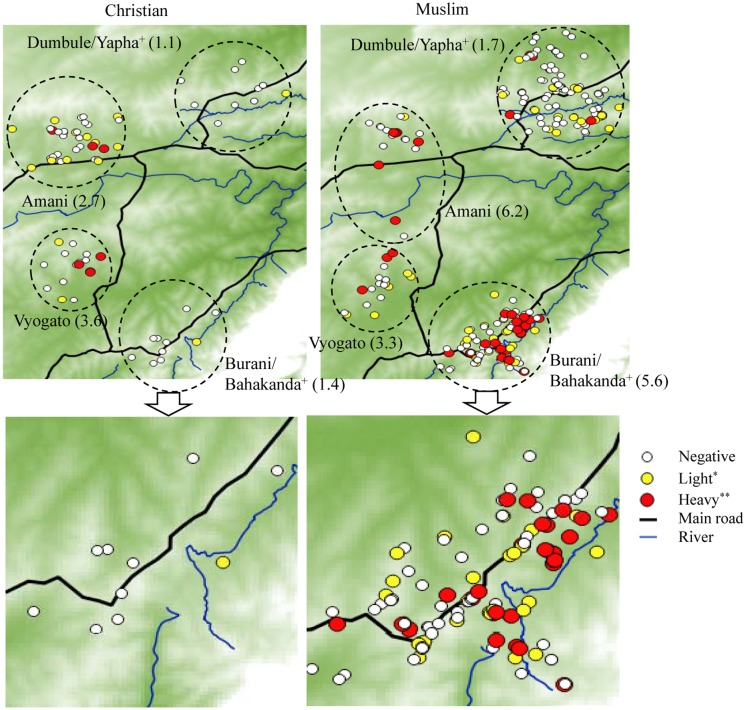
Spatial distribution of the intensity of *S*. *haematobium* infection in the study area. The intensity of infection was relatively high among Muslims compared to Christians despite participants sharing locality of residence. It is evident the intensity of infection is not related to the house proximity to the river. “Plus sign” schools combined because of overlap in the distribution of their populations in the study area. “Single asterisk” 1–49 eggs/10 ml urine, “double asterisks” ≥50 eggs/10 ml urine. The numbers in parentheses indicate the geometric mean of the number of eggs in each school based on religion.

The spatial distribution of the intensity of *S*. *haematobium* infection in the study area is illustrated in Fig 2. Generally, the geometric mean intensity (indicated in parentheses) was high among Muslims compared to Christians in all schools except Vyogato. The percentage of *S*. *haematobium* positive cases among Muslims (34.6%) was higher than positive cases among Christians (27.8%) but not significant (χ^2^ = 0.99044, P = 0.3196).

**Fig 3:**
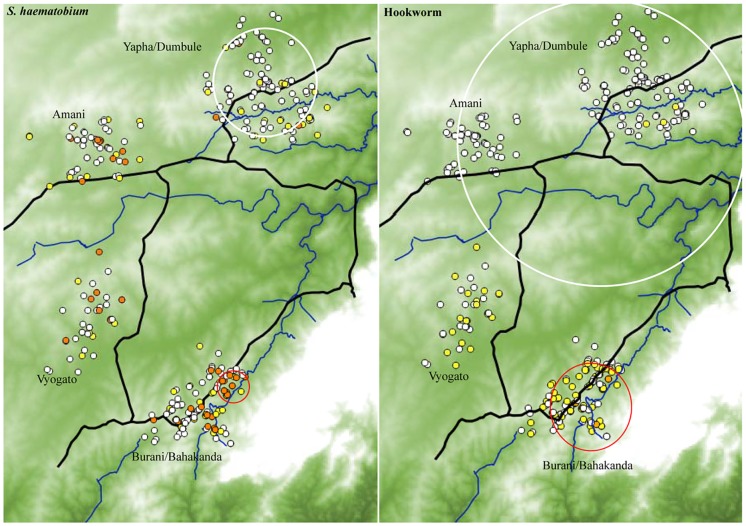
Clustering of *S*. *haematobium* and hookworm infections in the study area. The intensity was expressed as log_10_ (N + 1). In the left panel, *S*. *haematobium* was categorized based on WHO guidelines as: negative, light (1–49 eggs/10 ml urine) and heavy (≥50 eggs/10 ml urine) represented by white, yellow and red dots respectively. The red and white cycles show high and low risk clusters respectively. In the right panel, hookworm was grouped into negative, light (1–1999) and moderate (2000–3999) indicated by white, yellow and brown dots respectively. High risk cluster shown by red circle while the large white cycle represents the low infection cluster.

Spatial analysis revealed clustering of *S*. *haematobium* infection. A high risk cluster including 14 children with a radius of 620 meters was identified in Burani/Bahakanda. The mean of log (N+1) transformed egg count was 4.46 and 0.96 inside and outside the cluster respectively *P* = 0.001. A low infection risk cluster of radius 3050 meters including 102 children in Yapha and Dumbule was found. The mean inside 0.24 while the outside mean was 1.41, *P* = 0.011 (Fig 3).

### Spatial distribution and factors associated with the intensity of hookworm infection

The outcome of bivariate analysis on the association between the intensity of hookworm infection and the potential risk factors with the exclusion of school effects (NB-GLM) is displayed in Table 5. Participants who were dewormed more than one year prior to the study were more intensely infected than those dewormed within a year’s time *P* = 0.04951. Other factors which showed a significant association with high infection risk were; SES, latrine availability, religion and main source of drinking water. There was a significant low infection risk among participants who were residing far away from the river.

**Table 5:** Bivariate negative binomial generalized linear model (NB-GLM) for intensity of hookworm infection among schoolchildren in Kwale, Kenya.

Parameter		Estimate	Std. Error	Z value	Pr(> | Z |)
Age	8–18 years	0.2381	0.1573	1.514	0.13
Sex	Boys	-0.3534	0.5373	-0.658	0.511
*S*. *haematobium* infection	Positive	0.9868	0.5658	1.744	0.0811
SES* (*Reference = Least poor)	Less poor	2.01375	0.82227	2.449	**0.0143**
	Poor	0.99243	0.81971	1.211	0.226
	Very poor	1.96882	0.84231	2.337	**0.0194**
	Most poor	-0.06043	0.82783	-0.073	0.9418
Last deworming	Over a year ago	1.194	0.6134	1.95	**0.04951**
Bathing in river/dam** (**Reference = Never)	1–2 times a week	-2.223	1.2178	-1.825	0.0679
	5–6 times a week	-2.521	1.4977	-1.683	0.0923
	Daily	-0.8499	0.6012	-1.414	0.1575
Religion	Muslim	1.9862	0.6431	2.088	**0.002**
Latrine	Present	1.1808	0.5578	2.117	**0.0343**
Drinking water source*** (***Reference = River)	Well	1.891	0.8027	2.356	**0.0185**
	Spring	-0.149	2.9244	-0.051	0.9594
	Tap	0.614	0.8028	0.765	0.4444
Shoes wearing habit	Never	-0.4209	0.5375	-0.783	0.434
Distance to the river	Far	-2.1489	0.5153	-4.17	**3.04E-05**

In the NB-GLMM on inclusion of school random factor, the effects of latrine, religion and source of drinking water became non-significant. SES, sex and distance to the river were significantly associated with the intensity of hookworm infection. Table 6 details the final predictors of hookworm infection risk.

**Table 6:** Negative binomial generalized linear mixed model (NB-GLMM) for intensity of hookworm infection among schoolchildren in Kwale, Kenya.

Parameter		Estimate	Std. Error	Z value	Pr(> | Z |)
Age	8–18 years	0.0766	0.3171	0.56	0.5765
Sex	Boys	-0.9996	0.467	-2.14	**0.03233**
SES* (*Reference = Least poor)	Less poor	2.6252	0.8072	3.25	**0.00115**
	Poor	0.1248	0.7509	0.17	0.86802
	Very poor	1.4673	0.8049	1.82	0.06831
	Most poor	2.147	0.8479	2.53	**0.01134**
Last deworming	Over a year ago	1.194	0.6134	1.95	0.05159
Religion	Muslim	0.0345	0.7136	0.05	0.61586
Latrine	Present	-0.5762	0.6788	-0.85	0.86298
Drinking water source** (**Reference = River)	Well	0.4025	0.8023	0.5	0.61586
	Spring	-0.3625	2.1003	-0.17	0.86298
	Tap	-0.9887	0.8237	-1.2	0.23002
Distance	Far	-1.9075	0.5697	-3.35	**0.0081**

In Fig 3, a significant high risk cluster for hookworm infection was singled out in Burani and Bahakanda (radius 2470 meters and included 103 children). The respective mean of log (N+1) transformed egg counts inside and outside was 3.07 and 0.42, *P* = 0.001. A low cluster of the intensity of hookworm infection was identified around Amani, Yapha and Dumbule schools. All children in the three schools except 14 children in Amani were included. The mean of log (N+1) transformed egg counts inside the low cluster was 0.095 while outside mean was 2.23, *P* = 0.001.

## Discussion

The current study demonstrates both *S*. *haematobium* and hookworm infections are significant public health problems in Kwale. Identifying local risk factors is essential for expediting disease control by targeting high-risk groups or by informing possible intervention strategies to stakeholders involved in helminthiasis control. The proportion of schoolchildren infected with *S*. *haematobium* was 33.2%. This result is consistent with recent reports preceding this study [35,36]. However, this frequency is lower than that in the 1980s [37,38], when the prevalence was >70% among school-aged children in Kwale. Among STHs, hookworm is the most common in Kwale, and this finding corroborates past studies [36,39].

Clustering of the density of *S*. *haematobium* infection was evident in the micro-geographical study. The proportion of children with heavy infection intensity was lower in Dumbule and Yapha compared to other schools in the study area. Focality of schistosomiasis even in small-scale geographical settings is a well-known phenomenon [13–17]. Notably, the two schools with lower infection density were located in a dry area compared to the other schools. Such environments are not suitable for the propagation of intermediate host snails of schistosomiasis.

In NB-GLMM analysis, children from Muslim households excreted large numbers of *S*. *haematobium* eggs. The school was included in the model as a random factor to adjust for environmental effects, with the assumption that children attending a given school were clustered around the school. On stratification of the study population by the school, the intensity of *S*. *haematobium* was consistently high among Muslims in all schools except Vyogato, where the infection intensity appeared to be similar both in Muslims and Christians. The high intensity of *S*. *haematobium* among Muslims compared to Christians is of great interest. A further investigation of the underlying religion determinants of our observed difference in the intensity of *S*. *haematobium* in this population based on religious affiliation is necessary. A past study in Kwale indicated Muslims had lower participation in control and related operational research for urogenital schistosomiasis and soil-transmitted helminthiasis by 50% compared to Christians [40]. We could not clarify the reason why Muslims only showed higher intensity of *S*. *haematobium* infection than Christians but not both intensity and prevalence. Health seeking behavior can be influenced by religious or cultural beliefs [41]. A few heavily infected individuals can maintain the transmission of schistosomiasis. To effectively control schistosomiasis, identifying and targeting such heavily infected individuals is critical. Furthermore, profiling such heavily infected individuals can help us understand the disease epidemiology in endemic regions. The density *S*. *haematobium* infection was high among children from households with low SES. This finding is in agreement with a former study [42]. The relationship between low SES and infection intensity could be attributed to the correlation between low SES and poor sanitation and inaccessibility to safe water. We did not observe a relationship between the intensity *S*. *haematobium* infection and sex, contrary to past studies [39,43,44] but in agreement with an earlier study in coastal Kenya [45]. Past studies also found association of *Schistosoma* spp. infection with proximity to open water sources [13–17], there was no relationship between the intensity of *S*. *haematobium* infection and house distance to the river in Kwale setting.

Cluster analysis revealed a low hookworm infection cluster around Amani, Dumbule and Yapha schools. Notably, these schools are located in a drier area compared to the rest of schools in the study site. Transmission of hookworm is least likely to be supported in such dry conditions.

The final predictors of hookworm infection risk were: SES, sex, and distance to the river. Higher intensity of hookworm infection was observed among the poorer categories i.e., the less and most poor compared to the least poor group. Poverty is associated with multiple factors such as the absence of concrete floors in home dwellings, inadequate sanitation and lack of access to ant-helminths. Such factors can promote hookworm infection risk [46]. The hookworm infection risk among boys was lower compared to girls. Our findings are contradictory to the past studies where higher infection has been observed in boys [39,47,48]. The risk of hookworm infection risk declined with increased residence distance from the river. This can be explained by the survival rate of the infective larval stage that depends on the presence of optimal soil humidity and temperature conditions [49]. The three schools with low hookworm risk were in a drier area with high mean distance of residence from the river. History of anthelmintic treatment was marginally associated with the intensity of hookworm infection in Kwale. Individuals are prone to reinfection especially when chemoprophylaxis is the only strategy for hookworm control. Hookworm infection density was lower among children who received anthelmintic medication within 1 year before our study. This is in agreement with a study in Uganda where hookworm infection intensity was lower among participants who reported anthelmintic treatment within the last 6 months [48]. To expedite the control of hookworm infection in our study setting, intensified preventive chemotherapy strategies, i.e., increased frequency and coverage, are necessary. Past studies observed an association between hookworm infection and age [50–53]. However, in this study, hookworm infection intensity was not associated with age.

We acknowledge some limitations of our study. First, we only inquired about river water contact not considering other potential sources of schistosomes. Second, we did not investigate the duration of contact with water infested with *Schistosoma* larvae. Quantifying contact activities with infested water is necessary to assess the contribution of water contact behavior to schistosomiasis in endemic regions [54]. Third, use of a questionnaire to gather information on past deworming history is subject to recall bias. Finally, the study participants could easily confuse other medications taken in the past to be anthelmintic treatment.

Both *S*. *haematobium* and hookworm infections showed micro-geographical heterogeneities in this Kwale community. To confirm and explain our observation of high *S*. *haematobium* risk among Muslims, further extensive investigations are necessary. The observed small-scale clustering of the *S*. *haematobium* and hookworm infections might imply less uniform strategies even at finer scale for efficient utilization of limited resources.

## Supporting information

S1 Checklist STROBE checklist. (DOC)

S1 File Generation of wealth index in SPSS. (DOCX)

S2 File Supporting data. (XLSX)

S1 Table Prevalence and intensity of helminthic infections among schoolchildren, Kwale. (XLSX)
