# Supplementary material for: Spatial distribution and risk factors of Schistosoma haematobium and hookworm infections among schoolchildren in Kwale, Kenya
Source: PLoS Negl Trop Dis. 2017 Sep 1;11(9):e0005872. doi: 10.1371/journal.pntd.0005872 (PMC5599053; doi:10.1371/journal.pntd.0005872)
Supplement: S1 File — (DOCX) [file pntd.0005872.s002.docx]

S1 File. Generation of wealth index in SPSS

Variables included:

1. Main house roofing material: (1) Grass (2) Tin cans (3) Corrugated Iron (4) Asbestos (5) Concrete (6) Tile
2. Main house floor material: (1) Earth (2) Wood plank (3) Polished wood (4) Linoleum (5) Tile (6) Cement
3. Main house wall material: (1) Mud (2) Cemented/blocks/bricks (3) Wood (4) Iron sheets
4. Average number of household members sharing a room for sleeping ----------------
5. Total land (in acres) owned by household ---------------------
6. Possession of:
7. Mobile phone () Yes () No
8. Radio () Yes () No
9. Television () Yes () No
10. Solar panel () Yes () No
11. Bicycle () Yes () No
12. Motor cycle () Yes () No

**Recording of variables was done as follows:**

1. Main house roofing material: 0 = Grass or Tin cans 1 = Corrugated Iron or Asbestos or Concrete or Tile
2. Main house floor material: 0 = Earth or Wood plank 1 = Polished wood or Linoleum or Tile or Cement
3. Main house wall material: 0 = Mud/Woo/Iron sheets 1 = Cemented/blocks/bricks
4. Average number of household members sharing a room for sleeping 0 = (> persons sharing a sleeping room) 1 = (≤ 3 persons sharing a sleeping room). This is based on average persons sharing a sleeping room which was 3.
5. Total land (in acres) owned by household 0 = (< 4 acres) 1 = (≥ 4 acres). This is based on average land size owned by household which was 4 acres.
6. Possession of various assets 0 = No 1 = Yes.

**SPSS PCA output**


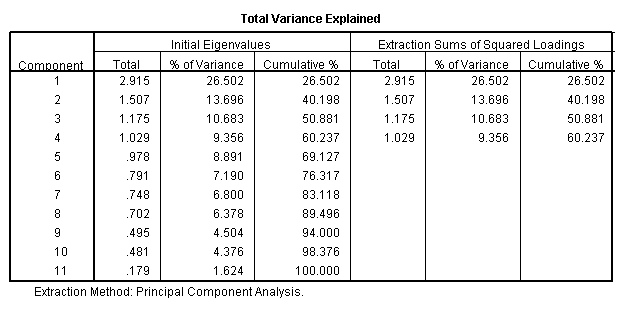


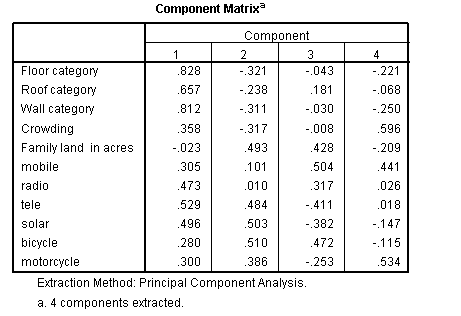


The first component was used for generation of wealth index since it accounted for the greatest variance. This variable had values ranging from -1.5797 to 2.10968. This continuous variable was converted into categorical variable in SPSS by ranking it into quintiles through case ranking. The least rank was assigned 1 while the highest rank was assigned 5.

These values were later renamed as

1 = Most poor

2 = Very poor

3 = Poor

4 = Less poor

5 = Least poor
